# The effectiveness of Promoting Alternative Thinking Strategies program: A meta-analysis

**DOI:** 10.3389/fpsyg.2022.1030572

**Published:** 2022-12-09

**Authors:** Jieping Shi, Alan C. K. Cheung, Aohua Ni

**Affiliations:** Department of Educational Administration and Policy, The Chinese University of Hong Kong, Hong Kong, Hong Kong SAR, China

**Keywords:** Promoting Alternative Thinking Strategies, social emotional learning, meta-analysis, systematic review, program effectiveness

## Abstract

Promoting Alternative Thinking Strategies (PATHS) is a widely-used social emotional learning program for preschool and elementary school students. The purpose of this review is to examine its effects, and explore the moderation effects of methodological and implementation features on intervention effectiveness. Using stringent inclusion criteria, 20 qualified studies and 177 effect sizes involving 30,454 participants were included. Results showed that the overall effect size of PATHS was 0.11. In particular, the effect size of PATHS on social emotional skills (ES = 0.16) was the largest compared with other outcome domains, including attitude or relations (ES = 0.08), emotional well-being (ES = 0.02), prosocial behaviors (ES = 0.04), conduct problems (ES = 0.06), and academic performance (ES = 0.05). PATHS had no different impact whether it was implemented in the universal or target contexts. Research design, sample size, and intervention dosage could moderate the effectiveness of PATHS significantly, and dosage was the predominant factor in determining the effects of PATHS. Policy and practical implications were discussed.

## Introduction

There is an increasing consensus on the crucial role of social emotional learning in student development among parents, teachers, and policy makers (Miyamoto et al., 2015). Social emotional learning refers to the processes through which “children and adults acquire and effectively apply the knowledge, attitudes, and skills necessary to understand and manage emotions, set and achieve positive goals, feel and show empathy for others, establish and maintain positive relationships, and make responsible decisions” (Collaborative for Academic Social Emotional Learning [CASEL], 2015, p. 5). Social emotional learning has been broadly implemented for decades from kindergarten to high school globally, and its effectiveness has been supported by a large number of randomized controlled trials and quasi-experimental studies (e.g., Jones et al., 2011; Crean and Johnson, 2013; Novak et al., 2017; Seyhan et al., 2019; Upshur et al., 2019). Though several previous reviews (e.g., Durlak et al., 2011; Sklad et al., 2012; Wigelsworth et al., 2016) have estimated the effects of social emotional learning intervention programs for young children, few have done so separately by one specific program using rigorous inclusion criteria.

The purpose of this review is to examine the effects of Promoting Alternative Thinking Strategies (PATHS) on preK-6 students. PATHS is one of the most widely-used social emotional learning programs for preschool and elementary school children. It was first designed by Greenberg and Kusche to improve social and emotional development for deaf children in 1982 (Greenberg and Kusché, 1993). It was later adapted and conducted as a universal social emotional intervention in mainstream classrooms. Since then, the objectives and content have continued to be expanded. All versions of PATHS emphasize common core components in the curriculum like self-control, emotional understanding and interpersonal problem solving (Greenberg et al., 2004). The primary focus is to reduce behavioral and emotional problems, improve academic performance and facilitate emotional process through promoting children’s social emotional development (Kusché and Greenberg, 2020). So far, PATHS has been translated into multiple versions in different languages and implemented for decades in different countries, such as Canada, the UK, Netherlands, Australia, Turkey, Croatia, Switzerland (Kusché and Greenberg, 2020).

The reasons for selecting PATHS are threefold: (1) wide reach of use. It was implemented in more than 20 countries and thousands of schools. For instance, over 250 schools delivered PATHS in the UK (The Paths Programme for Schools (Uk Version), n.d.); (2) high quality. It was identified as a “SELect” or high quality social emotional learning program by Collaborative for Academic Social Emotional Learning [CASEL] (2013, 2015) and Jones et al. (2017). It was rated as one of the 15 “Model Programs” among over 1,400 programs by Blueprints for Healthy Youth Development (The Paths Program, n.d.); (3) largest number of experimental studies. The What Works Clearinghouse (WWC) identified 35 PATHS studies, and we found over 50 experimental studies, which was the largest compared to other SEL programs. Therefore, the current review will choose PATHS to conduct an in-depth review and explore which features could moderate its effects.

## The PATHS program

### Theory of PATHS

The PATHS program is based on the Affective-Behavioral-Cognitive-Dynamic (ABCD) theoretical model, which underlines the developmental integration of affects, behaviors, and cognitions. It is generated from multiple psychological theories, including developmental social cognition, cognitive development theory, and attachment theory (Greenberg and Kusché, 1993). The premise of this model declares that “the child’s coping, as reflected in his or her behavior and internal regulation, is a function of emotional awareness, affective-cognitive control, and social-cognitive understanding” (Greenberg and Kusché, 1993). In addition, the ABCD model believes that different phases of development have different defenses from infancy to adolescence, and there are substantial changes from the previous phase to the next phase. From this development of defense mechanisms, it can be inferred that affective development occurs in advance of other modes of development in the process of individual growth (Greenberg et al., 2004). In other words, affective development should be paid attention to in the early stages of child growth due to its pioneering role. The ABCD model also highlights the crucial role of language development in personality development, indicating that it can incorporate with affective development and then process the previous emotional forms into linguistic forms (Greenberg and Kusché, 1993). This verbal mediation of affect should be cultivated in the lower grades of elementary school. Additionally, language development can also contribute to emotional management and behavioral control. In short, the ABCD model provides a general holistic and dynamic mechanism of individual maturation in terms of affects, behaviors, and cognitions, and illustrates the critical development stages of each function, which lays a solid foundation for the design and implementation of the PATHS curriculum.

Moreover, there are three additional theories related to the PATHS program. First, ecological theory provides another perspective from which to understand and facilitate the development of students. It explains that the child interacts with parents, teachers, peers, and others in different ecological settings and these settings affect each other (Bronfenbrenner, 1979). Particularly, teachers act as role models and have great influence on children’s social and emotional development during school years. Under this circumstance, PATHS is no longer restricted to the courses that teach children skills. It also emphasizes the creation of a positive and supportive class environment and school environment, and provides opportunities for students to apply the social emotional skills they have learned (Greenberg et al., 2004).

Second, the ABCD model of PATHS, like any other psychological model, must be consistent with the developmental neurobiology. There are two important mechanisms of brain organization, “vertical” control and “horizontal” control. “Vertical” communication and control involves the higher-order processing of emotions and actions in which the limbic system transfers the information from sensory-motor areas to the neocortical areas and then the neocortical areas modify impulses and send messages back to the limbic system. “Horizontal” control involves the interaction processing of the left hemisphere and the right hemisphere (Greenberg et al., 2004). If a child fails to achieve neocortical control by around the age of seven, “behavioral problems” will occur (Greenberg and Kusché, 1993). Therefore, based on these two kinds of communication and control, the PATHS program offers multiple strategies and materials to help a child manage emotions and control behaviors, like self-talk, feeling face cards, etc. (Greenberg et al., 2004).

Third, psychoanalytic education is highly related to the development of PATHS, which makes PATHS different from many other social emotional learning programs (Kusché and Greenberg, 2012). It highlights “positive teacher-student relationships, internalization of prosocial values, use of creativity, optimal educational and cognitive integration, appropriate expression (rather than repression) of affect, and learning as a process of joyful discovery” (Kusché and Greenberg, 2012). Children are no longer required to comply with external expectations, but are encouraged to actively participate and interact in the learning process (Greenberg et al., 2004). From this perspective, PATHS is enjoyable for both teachers and students to teach and learn (Kusché, 2002).

### Implementation of PATHS

The PATHS curriculum is a series of school-based comprehensive lessons which aims to improve social emotional skills, prevent emotional and behavioral problems, enhance academic performance, and reinforce a positive atmosphere in classrooms and schools. In general, it consists of five aspects, self-control, emotional understanding, positive self-esteem, relationships, and interpersonal problem-solving skills (Kusché and Greenberg, 2020). There are approximately 40 lessons in each grade from Pre-K to grade 6, involving the aforementioned domains with different levels of difficulty. It can be taught by teachers to regular classes as well as self-contained special education classes with the dosage of two to three times per week. It provided students with a variety of classroom activities, such as story, discussion, role-play, drawing, music, and painting. Moreover, it is important for teachers to encourage students to generalize the corresponding social emotional skills into real-life situations. For instance, students can integrate them into other academic subjects, or play games or communicate with their parents to strengthen the concepts and skills in the PATHS curriculum. In addition, teachers are required to participate in a short-term training workshop before starting the course, and can get additional technical support and consultation during the procedure of teaching courses.

## Previous relevant reviews

To the best of our knowledge, there is only one review focusing on the effects of the PATHS program. That review synthesized the published studies on PATHS in preschool settings, and found that preschool PATHS had a positive mild to moderate effect on children’s social emotional competence and little effect on problem behaviors (Stanley, 2019). Since there were only five studies included, the review looked at the effects of each study separately, instead of performing a meta-analysis. Two of the included studies did not have control groups (i.e., Gibson et al., 2015; Mihic et al., 2016), and one study had a control group of less than thirty participants (i.e., Hughes and Cline, 2015). Therefore, the findings of the effects of preschool PATHS in this review should be interpreted with much caution.

In addition to this preschool PATHS review, two other reviews of Second Step may be of some relevance to the current review because both programs consist of similar social emotional components. Moy and Hazen (2018) analyzed 24 studies in which Second Step was implemented as a Tier 1 intervention, and found its effects on knowledge of program content, prosocial outcomes and antisocial outcomes in independent group design studies were 0.77, 0.06, and −0.11, respectively. However, it employed a set of rather loose inclusion criteria, which might hinder the accuracy of the findings of the overall effects. For instance, 10 of the 24 included studies used a single-group repeated measures design, which might overestimate the effect sizes. The other review also conducted by Moy et al. (2018) synthesized 27 randomized controlled or quasi-experimental studies of Second Step from 1984 to 2016, indicating a large effect of 1.08 on program knowledge and two small effects of 0.19 and 0.22 on prosocial and antisocial behaviors. It also examined five factors which could moderate the effects of Second Step, but the corresponding analysis was limited. In short, these two reviews summarized the effects of another social emotional learning program Second Step, which supported the feasibility of examining a particular social emotional learning program in depth.

Finally, there are several reviews synthesizing the effects of multiple school-based social emotional learning programs (Payton et al., 2008; Durlak et al., 2011; Sklad et al., 2012; Wigelsworth et al., 2016; Blewitt et al., 2018; Corcoran et al., 2018; Yang et al., 2019; Murano et al., 2020). Compared to the reviews targeting one program, they involve diverse social emotional learning programs in terms of objectives, participants, durations, courses, assessments, etc., which could lead to a high heterogeneity of included studies. For instance, one widely-cited review by Durlak et al. (2011) summarized the effects of 213 school-based social emotional learning studies on Pre-K-12 students from 1955 to 2007. They found that social emotional learning could significantly promote social emotional skills (*ES* = 0.57, *k* = 68), attitudes toward self and others (*ES* = 0.23, *k* = 106), positive social behavior (*ES* = 0.24, *k* = 86), academic performance (*ES* = 0.27, *k* = 35) and reduce conduct problems (*ES* = 0.22, *k* = 112) and emotional distress (*ES* = 0.24, *k* = 49). In addition, four recommended practices of developing students’ skills (SAFE, including Sequenced, Active, Focused, Explicit) and program implementation could moderate the effectiveness of social emotional learning. In particular, SAFE criteria were too loose that most studies could achieve, hindering their further implications for policy and practice. Implementation problems significantly decreased social emotional learning outcomes, but the authors did not specify what those implementation problems were. Consequently, there was no meaningful suggestions for implementation, even though two moderators were effective. Sklad et al. (2012) analyzed 75 published studies about social emotional or behavioral programs from 1995 to 2008, and concluded that this kind of intervention could improve social emotional skills (*ES* = 0.70, *k* = 31), academic performance (*ES* = 0.46, *k* = 10), positive self-image (*ES* = 0.46, *k* = 8), prosocial behavior (*ES* = 0.39, *k* = 6), and decrease antisocial behavior (*ES* = 0.43, *k* = 39), mental disorders (*ES* = 0.19, *k* = 13) and substance abuse (*ES* = 0.09, *k* = 10). It also showed that the effect size of studies with 20 sessions or more on social skills were 0.24, whereas the effect size of studies with less than 20 sessions were 0.80, which seemed to be counterintuitive. Wigelsworth et al. (2016) also examined the effects of 89 universal school-based social emotional learning studies, and found small to medium effects on social emotional competence (*ES* = 0.53, *k* = 24), attitudes towards self (*ES* = 17, *k* = 9), prosocial behavior (*ES* = 0.33, *k* = 39), conduct problems (*ES* = 0.28, *k* = 40), emotional distress (*ES* = 0.19, *k* = 32), academic achievement (*ES* = 0.28, *k* = 15) and emotional competence (*ES* = 0.27, *k* = 14). Taylor et al. (2017) explored the follow-up effects of social emotional learning and found seven small positive effect sizes on social emotional skills, attitudes, positive social behavior, academic performance, conduct problems, emotional distress and drug use. In short, these four comprehensive reviews synthesized the effects of social emotional learning on multiple outcome domains at post-test and follow-up, and the classification of outcomes was almost the same. However, they failed to find any meaningful moderation effects for social emotional learning implementation, owing to the high heterogeneity of the included studies.

Moreover, there were another four reviews that only focused on the effects of preschool social emotional learning programs, and their categorization of outcomes was different from the above four reviews. The first one analyzed 63 studies involving children aged 2 to 6 years and pointed out that social emotional learning had positive effects on social competence, emotional competence, problem behaviors and emotions, self-regulation, and early learning outcomes, of which the effect sizes ranged from 0.18 to 0.54 (Blewitt et al., 2018). The second review summarized 29 studies for low-income children aged 3-5 years and obtained a small positive effect size of 0.24 on social emotional competence (Yang et al., 2019). The third one estimated the effects of universal and targeted social emotional learning programs in preschool. This review found that both universal and targeted social emotional learning could improve social emotional skills and reduce problem behaviors, with effect sizes of 0.34, 0.32, 0.44, and 0.50, respectively (Murano et al., 2020). The last one involved 11 preschool social emotional learning studies, but their effects and quality varied largely (Sabey et al., 2017). In addition, another review of social emotional learning may be also relevant. It concentrated on the effects of social emotional learning on academic performance, and found that social emotional learning could improve scores on reading, math and science with effect sizes of 0.25, 0.26, and 0.19 (Corcoran et al., 2018). Consequently, the results of these reviews were quite consistent, showing positive effects of social emotional learning on multiple outcomes.

In sum, there is a general consensus that social emotional learning has positively small to medium effects on multiple outcomes, including social emotional skills, attitudes, prosocial behavior, conduct problems, emotional distress, and academic performance. But these reviews did not reach a general consensus of the moderating effects of implementation features. Therefore, it is advisable to conduct an in-depth review of the effects of one particular social emotional program PATHS to explore which implementation factors works. First, this review will employ a set of rigorous criteria, which can result in a more convincing conclusion of PATHS. Second, both universal PATHS and targeted PATHS studies will be included in the current review, which provides the ability to compare the effects of PATHS in different contexts. Third, it will extract and identify multiple methodological features and implementation features of studies, and then examine their moderation effects. Particularly, the moderation effects in this review will be more convincing than other reviews of social emotional learning, since the treatments in the present corpus of studies are highly homogeneous, which can minimize the impacts of the intervention itself. Finally, it will update and enrich the results of previous reviews on PATHS or other related social emotional learning programs.

This review has three research questions.

(1) What is the overall effect of PATHS on students?
(2) Are there any differential effects of PATHS on particular subgroups of students?
(3) What features of included studies can moderate the effectiveness of PATHS?

## Materials and methods

### Searching procedures

In order to find all possible articles about the effects of PATHS, we employed the following three searching strategies. First, we used the term “Promoting Alternative Thinking Strategies” or “PATHS” and “impact” or “effect” or “effectiveness” or “evaluation” or “assessment” as Abstract to search the academic databases, including Web of Science, Proquest, ERIC, PsycINFO. Second, we searched the references in previous related reviews of PATHS or social emotional learning, which might provide additional studies. Third, we searched the relevant websites of PATHS to reduce publication bias, and found some empirical articles and reports about PATHS. In this way, some unpublished gray studies could be obtained as a supplement.

### Criteria for inclusion and exclusion

The criteria used to select eligible studies are set out below.

(1) It must be written in English.
(2) It was published before December 31, 2020.
(3) It must focus on the effects on students. Studies only focused on the effects of PATHS on teachers were excluded (e.g. Bierman et al., 2013; Pas et al., 2015; Domitrovich et al., 2016; Berg et al., 2017).
(4) Only PATHS must be implemented for the treatment group. If the treatment group combined PATHS and other programs, the study was excluded (e.g. Ialongo et al., 2019; Bradshaw et al., 2020; Hart et al., 2021).
(5) It must have a control group. Studies without control group was excluded (e.g. Kelly et al., 2004; Kam et al., 2011; Raynor, 2011; Faria et al., 2013; Gibson et al., 2015; Mihic et al., 2016; Wilson, 2016; Humphrey et al., 2018). Further, if the control group had other social emotional learning components, the study was excluded (e.g. Schonfeld et al., 2012).
(6) At least two teachers and 30 students were required in each of treatment groups and control groups to reduce the potential interference of teachers’ effectiveness. Studies without enough participants were excluded (e.g. Greenberg and Kusche, 1998; Bardon et al., 2008; Howe, 2013; Hughes and Cline, 2015; McDaniel et al., 2021).
(7) It must be of initial equivalence at pre-tests in terms of outcome measurements. In other words, the baseline difference between treatment group and control group must be less than 0.25 standard deviation, which is suggested by What Works Clearinghouse. If a study didn’t have equivalent baseline scores or didn’t report whether baseline scores were equivalent, it was excluded (e.g. Kam et al., 2003; Curtis and Norgate, 2007; Saltali and Deniz, 2010; Arda and Ocak, 2012; Fishbein et al., 2016).
(8) It must have enough quantitative statistics to calculate effect sizes.

**FIGURE 1:**
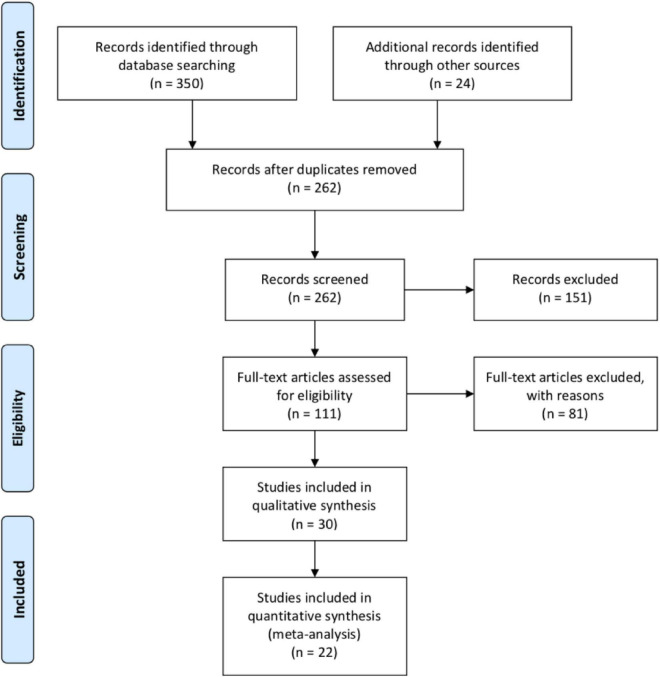
Flow chart of searching procedures.

In sum, there were 22 studies in the final sample, but only 20 of them had effect sizes at post-tests. Even though 30 articles were eligible, eight articles were partially duplicated because they used the same samples as other studies (i.e. Bierman et al., 1999a,^2002^, ^2007^; Malti et al., 2011; Sheard et al., 2013; Humphrey et al., 2015, 2016; Panayiotou et al., 2020). The flow chart shows the specific searching procedures step by step (Figure 1).

### Coding

Study coding mainly contained two parts. First, research features were coded as categorical moderators, namely: (1) research design, studies were coded as quasi-experiment or randomized controlled trial; (2) sample size, the numbers of participants in each study were coded as large (*N* > 1,000) or small; (3) grade level, studies were coded as Pre-K or elementary; (4) social economic status, studies were coded as low, middle, mixed or unknown according to the predominant social economic status of participants; (5) duration, the studies were coded as one year or more than one year; (6) dosage, studies were coded as standard if the interventions were implemented two or three times per week as recommended by the program. If the studies did not reach the standard dosage, they were coded as low; (7) intervention types, studies were coded as universal if the program was delivered for all students and as targeted if the program was offered to some specific students who needed additional support. Second, the outcomes of each study were categorized into six domains, namely social emotional skills, attitudes or relations, emotional well-being, prosocial behaviors, conduct problems, and academic performance. This classification approach is consistent with several previous reviews of social emotional learning (Payton et al., 2008; Durlak et al., 2011; Sklad et al., 2012; Wigelsworth et al., 2016). Two researchers coded the studies separately, and their coding reliability was 96%. Any disagreements about coding were solved through discussion.

### Statistical analysis

In the current review, standardized mean difference was used to explain the effects of PATHS on multiple outcomes for the included studies. Furthermore, all scores were transformed into the same direction, positive scores represented that the treatment group had better performance than the control group regardless of the outcome domains. For the studies with more than one effect sizes on different outcomes, we followed the suggestions of Borenstein et al. (2011) to calculate the synthetic effect size for each included study. As to the statistical analysis of the body of included studies, Hedge’s g was employed to attenuate the impacts of sample size, and the effect sizes of each study were weighted based on the inverse variance (Borenstein et al., 2011). Subgroup analysis and meta-regression were conducted to examine the effects of methodological factors and implementation features. Finally, the software Comprehensive Meta-Analysis version 3 was used in the current review.

## Results

### Overall effects

**FIGURE 2:**
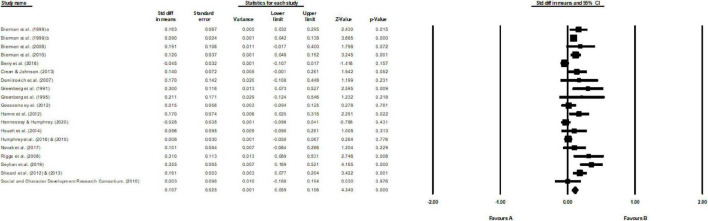
Forest plots of included studies.

A total of 22 studies met the inclusion criteria. However, two of these studies only reported effect sizes at follow-up rather than post-tests (i.e. Kam et al., 2004; Malti et al., 2011; Averdijk et al., 2016). Therefore, this review consisted of 20 qualified studies with 177 effect sizes, involving 30,454 participants (15,743 from treatment group, 14,711 from control group) from preschools to elementary schools. A brief description of the 20 included studies was shown in Table 1 and Figure 2. The result of the random model showed that the overall effect size of PATHS was 0.11 (*k* = 20), indicating a significantly positive effect. Moreover, the effect sizes of primary studies were of moderately high heterogeneity (*Q* = 60.61, *df* = 19, *p* < 0.05; *I*^2^ = 68.65%), which showed that the impacts of multiple PATHS programs were somewhat similar. Compared to the comprehensive reviews with 75% or higher *I*^2^, the heterogeneity of the current review was relatively low, indicating that restraining the various social emotional learning interventions to PATHS was an effective approach to reduce heterogeneity. Additionally, in order to examine whether there were any outliers that may bias the results, a “one study removed” operation was performed. After removing each study in turn, the effect sizes of the remaining studies ranged from 0.09 to 0.12, which lay in the 95% confidence interval from 0.06 to 0.16. In other words, the overall effect size was convincing because it would not change even if any one study was deleted.

**TABLE 1:** A brief description of included studies about PATHS.

References	Type	Design	Sample	Grade	SES	Duration	Dosage	Country	Overall ES
Bierman et al., 1999a,^2002^, ^2004^, ^2007^	Pub	Rct	Small	Ele	Mid	More	Standard	USA	0.163
Bierman et al., 1999b	Pub	Rct	Large	Ele	Low	1 year	Standard	USA	0.090
Bierman et al., 2008	Pub	Rct	Small	Pre	Mixed	1year	Low	USA	0.191
Bierman et al., 2010	Pub	Rct	Large	Ele	Low	More	Standard	USA	0.120
Berry et al., 2016	Pub	Rct	Large	Ele	Mixed	More	Low	Other	–0.045
Crean and Johnson, 2013	Pub	Rct	Small	Ele	Low	More	Low	USA	0.140
Domitrovich et al., 2007	Pub	Qed	Small	Pre	Low	1 year	Low	USA	0.170
Greenberg et al., 1991	Un	Qed	Small	Ele	NA	1 year	Standard	USA	0.300
Greenberg et al., 1995	Pub	Qed	Small	Ele	Mid	1 year	Standard	USA	0.211
Goossens et al., 2012	Pub	Qed	Large	Ele	NA	More	Low	Other	0.015
Hamre et al., 2012	Pub	Rct	Small	Pre	Low	1 year	Low	USA	0.170
Hennessey and Humphrey, 2020	Pub	Rct	Large	Ele	Mid	More	Low	Other	–0.028
Hsueh et al., 2014	Un	Rct	Small	Pre	NA	1 year	NA	USA	0.096
Humphrey et al., 2016, 2018	Un	Rct	Large	Ele	Mid	More	Low	Other	0.008
Johannes, 2003	Un	Qed	Small	Ele	NA	1 year	Low	USA	0.120
Novak et al., 2017	Pub	Rct	Small	Ele	NA	more	Standard	Other	0.101
Riggs et al., 2006	Pub	Qed	Small	Ele	NA	1 year	Standard	USA	0.310
Seyhan et al., 2019	Pub	Qed	Small	Pre	Mid	1 year	Standard	Other	0.355
Sheard et al., 2012, 2013	Pub	Qed	Large	Ele	Mid	More	NA	Other	0.181
Social Character Development Research Consortium [SACD], 2010	Un	Rct	Small	Ele	Low	More	Low	USA	0.003

Pub represents published studies, un represents unpublished studies; Rct represents randomized controlled trial, Qed represents quasi-experimental design; Pre represents preschool, Ele represents elementary school; 1 year represents one year or less, More represents more than one year.

Moreover, if the outcomes were categorized into six domains according to the classification in previous reviews, the effects varied (Table 2). Note that these outcomes were interrelated (Corcoran et al., 2020). In particular, PATHS had a small but significant positive effect on social emotional skills (*ES* = 0.16, *k* = 16), which was higher than the overall effect size. Nevertheless, the effects on other aspects were nearly negligible, including attitude or relations (*ES* = 0.08, *k* = 7), emotional well-being (*ES* = 0.02, *k* = 4), prosocial behaviors (*ES* = 0.04, *k* = 6), conduct problems (*ES* = 0.06, *k* = 14) and academic performance (*ES* = 0.05, *k* = 12). Moreover, the differences of effects among multiple domains were consistent with previous reviews (Durlak et al., 2011; Sklad et al., 2012; Wigelsworth et al., 2016), in which the effects on social emotional skills were substantially larger than those of the other outcomes. Therefore, the main outcome of PATHS was to promote social emotional skills, whereas the effects on reducing problem behaviors, improving academic performance, etc., were trivial.

**TABLE 2:** The overall effect sizes of PATHS.

Domain	Number of ES (n)	Number of studies (k)	ES	Standard error	Lower limit	Upper limit
Overall	177	20	0.11	0.02	0.06	0.16
Follow-up	58	5	0.10	0.06	–0.01	0.21
Social emotional skills	57	16	0.16	0.05	0.07	0.25
Attitude or relations	15	7	0.08	0.04	0.00	0.16
Emotional well-being	8	4	0.02	0.04	–0.07	0.11
Prosocial behaviors	11	6	0.04	0.02	0.01	0.08
Conduct problems	50	14	0.06	0.02	0.01	0.11
Academic performance	36	12	0.05	0.02	0.01	0.09

As to the follow-up effect sizes, there were only 5 studies and 58 effect sizes. The follow-up periods ranged from one to seven years. Results showed that the effect size of PATHS at follow-up was 0.10, which was marginally significant (*p* = 0.07). In other words, the PATHS program had a small and marginally significant long-term effect size, which might be a function of the small number of included studies. Note that the following analyses only focused on the post-test effect sizes owing to the limited number of follow-up studies.

### Publication bias

**FIGURE 3:**
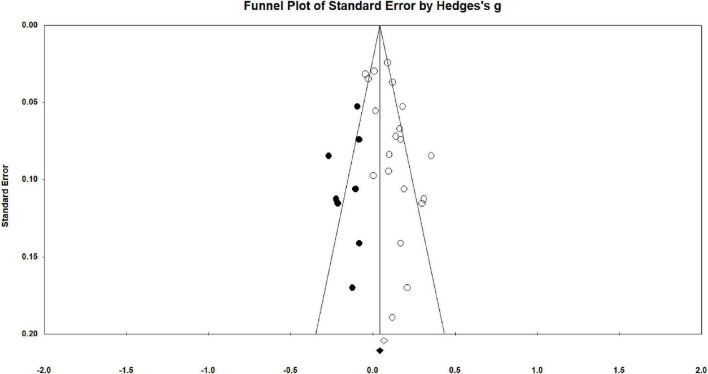
Funnel plot.

The publication bias was examined by multiple approaches. First of all, the result of the Classic fail-safe N test showed that 247 missing studies were needed to make the p-value become insignificant and the true effect become zero (*Z* = 7.16, *p* < 0.05). Second, the result of Orwin’s fail-safe N test explained that 115 missing studies were required if the trivial value was set to the 0.01 level. Both results showed that there was no publication bias because the number of missing studies was too large to be achieved. Additionally, the differences in effect sizes between published and unpublished studies were also examined. The results of the subgroup analysis showed that there was no difference between these two kinds of studies (*p* = 0.43 > 0.05; Table 3), which also partially supported the absence of publication bias. The funnel plot was shown in Figure 3.

**TABLE 3:** Subgroup analysis.

Study features	Effect size and 95% confidence interval					Test of heterogeneity		
		
	Number of studies	Point estimate	Standard error	Lower limit	Upper limit	*Q*-value	*df* (Q)	*P*-value
**Overall effect size**								
Random	20	0.11	0.02	0.06	0.16	60.64	19	0.00
**By publication**								
Published	15	0.12	0.03	0.06	0.17			
Unpublished	5	0.07	0.05	–0.03	0.17			
Total between	20					0.62	1	0.43
**By type**								
Universal	19	0.10	0.03	0.06	0.15			
Targeted	5	0.17	0.10	–0.02	0.36			
Total between	24					0.44	1	0.51
**By study design**								
QED	8	0.20	0.05	0.10	0.30			
RCT	12	0.07	0.02	0.02	0.12			
Total between	20					5.46	1	0.02
**By sample size**								
Small (≤1000)	13	0.17	0.03	0.12	0.23			
Large (>1000)	7	0.05	0.03	–0.01	0.10			
Total between	20					10.66	1	0.00
**By grade level**								
Preschool	5	0.20	0.05	0.11	0.29			
Elementary	15	0.08	0.03	0.03	0.13			
Total between	20					5.23	1	0.02
**By socioeconomic status**								
Low	6	0.10	0.02	0.07	0.14			
Middle	6	0.13	0.05	0.02	0.23			
Mixed	2	0.05	0.12	–0.18	0.28			
Unknown	6	0.13	0.05	0.03	0.24			
Total between	20					0.63	3	0.89
**By duration**								
One year	10	0.19	0.04	0.11	0.26			
More than one year	10	0.06	0.03	0.00	0.11			
Total between	20					7.50	1	0.01
**By dosage**								
Standard	8	0.17	0.03	0.10	0.24			
Low	10	0.03	0.03	–0.02	0.08			
NA	2	0.16	0.05	0.07	0.25			
Total between	20					12.78	2	0.00

### Subgroup analysis

Subgroup analysis was conducted to explore whether the PATHS program had differential impacts when it was delivered to different student groups. The intervention effects were classified as universal or targeted based on the characteristics of the participants. Since four studies included both universal and targeted effects, the subgroup analysis here was based on subgroup level instead of study level. Results showed that the effect size of targeted participants was slightly larger than that of universal participants, but it was not significant (*p* > 0.05). Hence there was not sufficient evidence to support that the PATHS program had significantly higher effects on targeted participants. One possible reason is that the number of effect sizes on targeted participants was too small, and another is that the characteristics of targeted participants were rather diverse.

### Moderators

Even though the heterogeneity of the included studies was not too high, the methodological features and implementation features of the studies were still examined as moderators in order to explore the factors which may have had an influence on the intervention effectiveness. Six features, namely research design, sample size, grade level, social economic status, duration, and dosage, were extracted and examined separately and simultaneously. Since the number of included studies was small, especially when the outcomes were classified into six domains, subgroup analyses and meta-regression were conducted on all outcomes instead of each domain.

#### Research design

Research design is an important factor that may affect the effect sizes of studies. In general, quasi-experimental studies have higher effect sizes than randomized controlled ones (Cheung and Slavin, 2016). In this review, the effect size of quasi-experimental studies (*ES* = 0.20, *k* = 8) was approximately three times that of randomized controlled studies (*ES* = 0.07, *k* = 12) and the difference between them was statistically significant (*p* < 0.05). Therefore, this result was consistent with previous studies, in which the research design could moderate the intervention effectiveness.

#### Sample size

Sample size is another factor which may moderate the effectiveness. Generally, studies with large sample sizes tend to have lower effect sizes (Slavin and Smith, 2009; Cheung and Slavin, 2016). In this review, the effect size of small studies (*ES* = 0.17, *k* = 13) was nearly four times that of large studies (*ES* = 0.05, *k* = 7), which was also significant (*p* < 0.05). In particular, the effect size of studies with more than 1,000 participants was only 0.05, which was not significantly different from zero (*p* > 0.05). Therefore, the large studies had almost negligible effect sizes, while the small studies had substantially positive effect sizes.

#### Grade level

As to the grade level, it may also moderate the effects of social emotional interventions (Sklad et al., 2012; Corcoran et al., 2018). The primary studies in this review were classified as preschool or elementary studies. Results explained that preschool studies (*ES* = 0.20, *k* = 5) did have higher effect sizes than elementary ones (*ES* = 0.08, *k* = 15), which was statistically significant (*p* < 0.05).

#### Social economic status

The predominant social economic status of participants may also have an influence on the intervention effects. In this review, the primary studies were coded as low, middle, mixed and unknown. The results showed that there was no significant difference among these four categories (*p* > 0.05). For instance, the effect size of studies with low socioeconomic background samples and the effect size of studies with middle socioeconomic background samples were both around 0.10. The difference between them was minimal.

#### Duration

The duration of intervention was tested as an indicator of effect sizes. The duration of the included studies was classified either as one year and more than one year. Results indicated that there was significant difference between these two categories (*p* < 0.05). Particularly, the effect size of studies which were implemented one year or less (*ES* = 0.19, *k* = 10) was about three times that of studies which were implemented for more than one year (*ES* = 0.06, *k* = 10).

#### Dosage

The relationship between dosage and intervention effectiveness was also examined. Since the PATHS curriculum is required to be taught two to three sessions per week in each grade, studies that took less than two sessions per week were marked as low dosage. Otherwise, they were marked as standard dosage. Results showed that the effect size of studies with standard dosage (*ES* = 0.17, *k* = 8) was approximately five times that of studies with low dosage (*ES* = 0.03, *k* = 10), which was statistically significant (*p* < 0.05). Specifically, the effect size of studies with low dosage was not significantly different from zero, indicating that low dosage would make the outcomes negligible, even though they used the same curriculum.

#### Meta-regression

Overall, only the social economic status of the six moderators was not significantly related to intervention effectiveness. Consequently, the other five moderators, namely research design, sample size, grade level, duration, and dosage, were those involved in the meta-regression. The aim of conducting meta-regression was to examine the effects of each factor simultaneously, since they could significantly moderate the intervention effects independently. The results of the random-effects model showed that three of the six covariates were statistically significant (Table 4). The variable of research design was statistically significant after controlling for other covariates (*Coefficient* = −0.09, *CI* = [−0.16 - −0.01], *p* < 0.05), indicating that the effect size of quasi-experimental studies was 0.09 higher than the effect size of randomized controlled studies. The variable of sample size was also significant (*Coefficient* = 0.08, *CI* = [0.00-0.15], *p* < 0.05), which illustrated that the studies with small sample size had a higher effect size of 0.08 than the large ones. The variable of dosage was statistically significant (*Coefficient* = −0.12, *CI* = [−0.19 - −0.06], *p* < 0.05), in which studies with low dosage had 0.12 lower effect sizes compared to studies with standard dosage. The variables of grade and duration were no longer statistically significant in the meta-regression. In particular, the coefficient of dosage was the largest among the three significant covariates, indicating that dosage was the strongest factor that could moderate the effects of the PATHS program. In addition, adding dosage alone to the regression could explain 54% of the variance, indicating that dosage was a predominant predictor of PATHS effects.

**TABLE 4:** Meta-regression for overall effects.

Random effects	Coefficient	Standard error	95% lower	95% upper	*Z*-value	*P*-value
Intercept	0.181***	0.042	0.100	0.262	4.36	0.000
Design (RCT)	−0.088*	0.038	–0.162	–0.014	–2.33	0.020
Sample size (Small)	0.077*	0.038	0.003	0.151	2.04	0.041
Grade (Pre-kindergarten)	0.082	0.066	–0.048	0.211	1.23	0.218
Duration (more than one year)	0.010	0.037	–0.063	0.083	0.27	0.788
Dosage (low)	−0.122***	0.033	–0.187	–0.057	–3.68	0.000
Dosage (NA)	–0.045	0.061	–0.165	0.075	–0.73	0.465

In meta-regression, the number of studies was 20, and the number of effect size was 177.

*p < 0.05, **p < 0.01, ***p < 0.001.

## Conclusion and discussion

The objective of the current review was to examine the effects of PATHS program on students and investigate the moderation effects of methodological and implementation features. Collectively, 20 studies involving 30,454 participants from preschool to elementary school were included, indicating a significant, small and positive effect of PATHS on students at post-tests (*ES* = 0.11). As to the follow-up effects ranging from one year to seven years, only 5 studies were eligible, indicating a small positive effect (*ES* = 0.10). In particular, the effect on social emotional skills was largest (*ES* = 0.16), whereas the effects on other domains, including attitude or relations (*ES* = 0.08), emotional well-being (*ES* = 0.02), prosocial behaviors (*ES* = 0.04), conduct problems (*ES* = 0.06) and academic performance (*ES* = 0.05), were nearly negligible. Regarding the interpretation of effect size, we adopted Kraft’s benchmarks for causal studies of Pre-K-12 education interventions, which supposed that less than 0.05 was small, 0.05 to less than 0.20 was medium, and 0.20 or greater was large (Kraft, 2020). The current overall effect size of 0.11 was medium, and the effect sizes for multiple outcomes was small to medium.

Two points should be highlighted and further explained for these findings. First, the overall effect size obtained in this review was substantially smaller than previous ones. In particular, the overall effect sizes of previous reviews mainly ranged from 0.20 to 0.70 (Durlak et al., 2011; Sklad et al., 2012; Wigelsworth et al., 2016; Taylor et al., 2017; Blewitt et al., 2018; Corcoran et al., 2018; Yang et al., 2019; Murano et al., 2020), whereas the effect size of this review was only about 0.11. One possible reason for these differences could be due to the inclusion criteria. The inclusion criteria in the current review were more rigorous than those in previous reviews, especially in terms of research design, sample size and initial equivalence. For instance, more than 20 experimental studies involving PATHS were excluded in the current review since they did not meet our stringent inclusion criteria. These studies were more likely to overestimate the effects since they had small sample sizes or did not have control groups, contributing to the less positive results. Second, the distribution of effects on multiple outcome domains in this review was roughly consistent with the previous ones. Previous reviews showed that the effect sizes of social emotional learning on social emotional skills were substantially larger than those on other outcome domains, including attitude or relations, emotional well-being, prosocial behaviors, conduct problems and academic performance (Durlak et al., 2011; Sklad et al., 2012; Wigelsworth et al., 2016). In this review, the effect size of PATHS on social emotional skills was also higher than those on other aspects. Consequently, the core output of PATHS or other social emotional learning programs was to reinforce students’ social emotional skills. Other outcomes, such as promoting prosocial behaviors, reducing problem behaviors, improving academic performance, etc., were all secondary.

Regarding the methodological factors and implementation features that may moderate the effectiveness, the results of univariate subgroup analysis and meta-regression differed slightly. In the current review, six factors were extracted to explain the heterogeneity of PATHS studies, namely research design, sample size, grade level, social economic status, duration and dosage. The results of univariate subgroup analysis showed that five of them could moderate the intervention effectiveness significantly with the exception of social economic status. However, only three factors were still statistically significant when the five factors were included in the meta-regression simultaneously. In other words, when controlling for the covariables of grade level and duration, the variables of research design, sample size, and dosage could still significantly moderate the intervention effectiveness. In particular, the quasi-experimental studies had a higher effect size of 0.09 than the randomized controlled studies, the studies with less than 1,000 participants had a higher effect size of 0.08 than the larger ones, and the studies that implemented two to three PATHS sessions per week had a higher effect size of 0.12 than the studies with lower dosage. The impacts of research design and sample size were consistent with previous findings (Slavin and Smith, 2009; Cheung and Slavin, 2016). Furthermore, the impact of dosage on intervention effectiveness was the largest, indicating that dosage was the predominant factor in determining the overall practical effects of the PATHS curriculum. It is worth noting that, to our best knowledge, this is the first time that the variable of dosage has been introduced in a meta-analysis of social emotional learning and its significant effect has been supported.

As to the differential effects for particular subgroups, there was no significant difference between targeted groups and universal groups. In the current review, targeted groups referred to students in special education classes, low social economic background students or students with more serious problem behaviors. Even though the effect for targeted participants was slightly larger than that for universal participants, the difference did not reach a statistically significant level. Two previous reviews about social emotional learning also exhibited similar findings. For instance, Payton et al. (2008) and Murano et al. (2020) highlighted that the targeted social emotional interventions had higher effects than the universal ones for at-risk preschool students or students aged 5 to 13 years, separately. There are three reasons that may account for the insignificant difference of PATHS on different groups. First, the number of targeted effects was too small to reach a statistically significant level. Second, the characteristics of targeted participants varied substantially, making it difficult to draw a general conclusion. Third, the interventions in different targeted studies were not the same. Some studies only implemented PATHS curriculum and examined the effects of some particular subgroups separately, whereas some studies carried out extra individual or small group consultation for targeted subgroups beyond the common PATHS curriculum. Consequently, the difference in effects between targeted students and universal students was not significant, even if there seemed to be a higher effect for targeted subgroups.

One key contribution of the current review was the findings on the predominant moderation effect of dosage on the impacts of PATHS. The reason for choosing dosage as one implementation feature was that most primary studies reported the frequency of conducting the curriculum and it had a good distinguishing feature among studies. If a study conducted 2 to 3 lessons per week as recommended by PATHS manual, it was considered as standard dosage. Conversely, if it failed to reach the dosage of two times per week, it was regarded as low dosage. This operational definition was very concise and easily accessible, and clearly divided primary studies into two categories, except for two studies that did not report relevant information. In contrast, other implementation features, such as quality, were difficult to extract due to limited information in most experimental studies. This distinguishing feature of dosage was partially supported by Hennessey and Humphrey (2020), who explained that distinct implementation profiles mainly differed in the levels of dosage irrespective of fidelity, quality, responsiveness and reach. Therefore, dosage is an excellent distinguishing feature of implementation, and its moderation effect on the outcomes of PATHS is supported in the current review. As mentioned above, dosage had a predominant influence on the effectiveness of PATHS among all moderators. If the PATHS curriculum was taught with the designed dosage of two to three times per week, the overall effect size was expected to be 0.17. However, if this dosage was not achieved, the overall effect size would drop to 0.03, which was not significantly different from zero. This positive relation between dosage and PATHS outcomes was consistent with the findings of some experimental studies (Faria et al., 2013; Schonfeld et al., 2015). In short, insufficient dosage would make an otherwise effective project ineffective.

This finding has potential policy and practical implications. In recent decades, social emotional learning programs have been increasingly carried out, but there is no doubt that academic performance still dominates in schools. Social emotional learning is often considered to be supplementary in schools (e.g., Murray et al., 2014; Gaspar et al., 2018; Seabra-Santos et al., 2018). Even if some schools do conduct curriculum-based social emotional learning programs, it is difficult to guarantee the dosage of courses (Bierman et al., 2008; Hamre et al., 2012; Berry et al., 2016; Humphrey et al., 2016; Hennessey and Humphrey, 2020). However, social emotional skills are at least as important as cognitive skills in predicting personal achievement in the future (Heckman et al., 2006). Social emotional learning, including PATHS, could not only cultivate social emotional skills but also indirectly improve students’ academic performance more or less (Durlak et al., 2011; Sklad et al., 2012; Wigelsworth et al., 2016). Therefore, the importance of social emotional learning should arouse more attention from policy makers, teachers and parents. One review about social emotional learning recommended that it should be conducted as regular curriculum rather than supplementary activities (Shi et al., unpublished^1^). The current review further suggests that the social emotional learning curriculum should be implemented with sufficient dosage to maintain effectiveness. After all, if the dosage does not achieve the recommended level, the overall effect size would become negligible even for a well-designed and high-quality program like PATHS. Note that the recommended dosage by PATHS might not be suitable for other social emotional learning programs, which should be taken seriously.

Some limitations in the current review need to be mentioned. First, since the number of studies involving subgroup effects (e.g., gender, race, etc.) was too small, we were not able to examine the differential effects of PATHS on these variables. Further experimental studies are expected to pay more attention to the differential effects of subgroups with special characteristics. Second, there were multiple informants for different measurement tools, including teachers, students, parents, and task observers. The informant might affect the effect sizes, but it was not included as a moderator, because one study might have more than one informant, which made it difficult to be analyzed on the study level. Since the number of included studies was limited, too many moderators may not be appropriate. Hence, we only selected some core moderators in the current review, which may omit other important moderators and bias our results. Finally, the effects of dosage on other social emotional learning programs need further exploration. The substantial effects of dosage should be interpreted with caution due to the limited number of studies. Further research may consider exploring a generally effective dosage cut-off point for social emotional learning, which would be meaningful for educational practice. Overall, the current review chose PATHS as a representative of social emotional learning, and found that dosage was a predominant predictor to the effectiveness of PATHS curriculum.

## Data availability statement

The original contributions presented in this study are included in the article/Supplementary material, further inquiries can be directed to the corresponding author.

## Author contributions

JS and AC performed the searching procedure, analytic calculations, and the final version of the manuscript. AN contributed to the revised version and the final version of the manuscript. All authors contributed to the article and approved the submitted version.

## Footnotes

1 Shi, J., Cheung, A. C. K., and Ni, A. (2022). Effective school-based social emotional learning programs in Pre-K-12 classrooms: A meta-analysis. Unpublished manuscript.
