# Supplementary material for: The effectiveness of Promoting Alternative Thinking Strategies program: A meta-analysis
Source: Front Psychol. 2022 Dec 9;13:1030572. doi: 10.3389/fpsyg.2022.1030572 (PMC9780599; doi:10.3389/fpsyg.2022.1030572)
Supplement: Supplementary file 1 [file Table_1.DOCX]

**Table S1**

List of included PATHS studies

| **Study** | **Design/**  **Sample size** | **N** | **SES or Sample Characteristics** | **Duration** | **Dosage/ Implementation of PATHS** | **Outcomes** | **Informant** | **Study ES** | **Target**  **ed ES** |
| --- | --- | --- | --- | --- | --- | --- | --- | --- | --- |
| Berry et al. (2016) | RCT(L) | 56 schools (28E, 28C) 4004 students (2203E, 1801C) | mixed; mainstream primary schools in a large city, Birmingham; UK | 2 years; K,1 | low; only taught 26 of 47 lessons | SDQ-conduct problems | teacher | -0.16 |  |
|  |  |  |  |  |  | SDQ-emotional difficulties | teacher | -0.06 |  |
|  |  |  |  |  |  | SDQ-hyperactivity | teacher | -0.09 |  |
|  |  |  |  |  |  | SDQ-peer relationships | teacher | 0.03 |  |
|  |  |  |  |  |  | SDQ-prosocial behaviors | teacher | 0.16 |  |
|  |  |  |  |  |  | PTRS-emotion regulation | teacher | -0.18 |  |
|  |  |  |  |  |  | PTRS-prosocial behavior | teacher | -0.06 |  |
|  |  |  |  |  |  | PTRS-social competence | teacher | -0.11 |  |
|  |  |  |  |  |  | PTRS-aggressive behavior | teacher | -0.02 |  |
|  |  |  |  |  |  | PTRS-internalizing/withdrawn | teacher | -0.05 |  |
|  |  |  |  |  |  | PTRS-inattention-hyperactivity | teacher | -0.04 |  |
|  |  |  |  |  |  | PTRS-impulsivity-hyperactivity | teacher | -0.03 |  |
|  |  |  |  |  |  | PTRS-peer relations | teacher | -0.08 |  |
|  |  |  |  |  |  | PTRS-relational aggression | teacher | -0.05 |  |
|  |  |  |  |  |  | PTRS-learning behaviors | teacher | -0.01 |  |
|  |  |  |  |  |  | PTRS-academic performance | teacher | 0.03 |  |

| Bierman et al. (1999) a & (2002) & (2004) & (2007) | RCT(S) | 54 schools 401 classrooms (191E, 210C) 891 students (445E, 446C) | mid; high-risk (top 10% on behavior problems); 35% lowest SES; 51% AA, 47% EA, 2% others; USA | 1 year; 1 | high; 2-3 sessions per week | ERQ-emotion recognition | task |  | 0.54 |
| --- | --- | --- | --- | --- | --- | --- | --- | --- | --- |
|  |  |  |  |  |  | IEE-emotion coping | task |  | 0.25 |
|  |  |  |  |  |  | SPS-social problem solving | task |  | 0.33 |
|  |  |  |  |  |  | HIWC-hostile attributions | task |  | 0.05 |
|  |  |  |  |  |  | HIWC-aggressive retaliation | task |  | 0.23 |
|  |  |  |  |  |  | WJPE-reading | task |  | 0.15 |
|  |  |  |  |  |  | SCS-social competence | parent |  | 0.04 |
|  |  |  |  |  |  | CBCL-externalizing behavior | parent |  | 0.04 |
|  |  |  |  |  |  | PDR-problem behavior | parent |  | 0.17 |
|  |  |  |  |  |  | TRF-externalizing behavior | teacher |  | 0.02 |
|  |  |  |  |  |  | CII/IRS-warmth/affection | observer |  | 0.23 |
|  |  |  |  |  |  | CII/IRS-noncompliance/aggression | observer |  | 0.22 |
|  |  |  |  | 3 years;  1-3 |  | TRF-externalizing behavior | teacher |  | 0.01 |
|  |  |  |  |  |  | PDR-problem behavior | parent |  | 0.15 |
|  |  |  |  |  |  | SPS-social problem solving | task |  | 0.14 |
|  |  |  |  |  |  | HIWC-hostile attributions | task |  | 0.14 |
|  |  |  |  |  |  | problem-free (cases) | combined |  | 0.21 |
|  |  |  |  | 5 years;  1-5 |  | social cognition and social competence problem domain | combined |  | 0.18 |
|  |  |  |  |  |  | peer deviance problem domain | combined |  | 0.11 |
|  |  |  |  |  |  | home and community problem domain | combined |  | 0.15 |
|  |  |  |  |  |  | school context problem domain | combined |  | -0.08 |

| Bierman et al. (1999) b | RCT(L) | 378 classrooms (198E, 180C) 6715 students | low; 55% FRL; 49% ethnic minority; USA | 1 year; 1 | high; 48.2 lessons per year, 2-3 sessions per week | TOCA-R-authority acceptance | teacher | 0.13 |  |
| --- | --- | --- | --- | --- | --- | --- | --- | --- | --- |
|  |  |  |  |  |  | TOCA-R-cognitive concentration | teacher | 0.04 |  |
|  |  |  |  |  |  | SHP-social competence | teacher | 0.10 |  |
| Bierman et al. (2008) | RCT(S) | 44 classrooms (22E,22C) 356 students | mixed; urban and rural areas in Pennsylvania; 17% H, 25% AA, 54% girls; USA | 1 year; pre | low; 33 lessons; 1.77 lessons and activities per week; high quality | EOWPVT-vocabulary | task | 0.15 |  |
|  |  |  |  |  |  | TOLD-grammatical understanding | task | -0.07 |  |
|  |  |  |  |  |  | TOLD-sentence imitation | task | -0.04 |  |
|  |  |  |  |  |  | TOPEL-emergent literacy skills-blending | task | 0.39 |  |
|  |  |  |  |  |  | TOPEL-emergent literacy skills-elision | task | 0.35 |  |
|  |  |  |  |  |  | TOPEL-emergent literacy skills-print awareness | task | 0.16 |  |
|  |  |  |  |  |  | ACES-emotion identification | task | 0.21 |  |
|  |  |  |  |  |  | ERQ-emotion recognition | task | 0.23 |  |
|  |  |  |  |  |  | CST-aggressive responses | task | 0.21 |  |
|  |  |  |  |  |  | CST-competent responses | task | 0.35 |  |
|  |  |  |  |  |  | CST-inept responses | task | 0.28 |  |
|  |  |  |  |  |  | SCS-social competence | parent | 0.09 |  |
|  |  |  |  |  |  | TOCA-R-aggression | parent | 0.13 |  |
|  |  |  |  |  |  | ALRAR-task orientation | task | 0.29 |  |
|  |  |  |  |  |  | ADHD-attention problems | parent | 0.10 |  |
|  |  |  |  |  |  | language/ communication | parent | 0.25 |  |
|  |  |  |  |  |  | reading activities | parent | 0.17 |  |
| Bierman et al. (2010) | RCT(L) | 2937 students | low; no high-risk students; around 47% FRL; USA | 3 years;  1-3 | high; 48, 40, 38 lessons were taught for G1,2,3; 2-3 times per week | TOCA-authority acceptance | teacher | 0.24 | 0.37 |
|  |  |  |  |  |  | TOCA-cognitive concentration | teacher | 0.12 | 0.24 |
|  |  |  |  |  |  | SHP-social competence | teacher | 0.34 | 0.70 |
|  |  |  |  |  |  | aggressive behaviors | peer | -0.03 |  |
|  |  |  |  |  |  | hyperactive behaviors | peer | 0.00 |  |
|  |  |  |  |  |  | prosocial behaviors | peer | 0.05 |  |
| Crean & Johnson. (2013) | RCT(S) | 14 schools (7E, 7C) 779 students (422E, 357C) | low; 51% Cau, 38% AA, 10% other; 43% met federal government defined income to household size poverty ratio; USA | 3 years;  3-5 | low; 34.8 lessons per year; 6 of 7 schools high quality, 1 school marginal quality | BASC2-aggression | teacher | 0.20 |  |
|  |  |  |  |  |  | BASC2-conduct problem | teacher | 0.15 |  |
|  |  |  |  |  |  | TCRS-acting out behavior problems | teacher | 0.24 |  |
|  |  |  |  |  |  | AS-aggression | student | -0.08 |  |
|  |  |  |  |  |  | FDBS-delinquent minor acts | student | -0.05 |  |
|  |  |  |  |  |  | VS-victimization at school | student | -0.09 |  |
|  |  |  |  |  |  | NBAAS-normative beliefs about aggression | student | 0.21 |  |
|  |  |  |  |  |  | SPSM-aggressive social problem solving | student | 0.27 |  |
|  |  |  |  |  |  | WWID-hostile attribution bias | student | 0.27 |  |
|  |  |  |  |  |  | WWID-aggressive interpersonal negotiation strategies | student | 0.28 |  |

| Domitrovich et al. (2007) | QED(S) | 20 classrooms (10E, 10C) 201 students | low; 47% AA, 38% EA, 10%H, 5% other; mean age 51 months; mean annual income for families 7039; USA | 1 year; pre | low; 30 lessons; once a week; high quality | DN-inhibitory control | task | -0.01 |  |
| --- | --- | --- | --- | --- | --- | --- | --- | --- | --- |
|  |  |  |  |  |  | PT-inhibitory control | task | -0.27 |  |
|  |  |  |  |  |  | LRAB-attention | task | 0.17 |  |
|  |  |  |  |  |  | CST-interpersonal problem solving | task | 0.08 |  |
|  |  |  |  |  |  | HSCS-Head Start competence | teacher | 0.46 |  |
|  |  |  |  |  |  | PKBS-social skills | teacher | 0.48 |  |
|  |  |  |  |  |  | HSCS-Head Start competence | parent | 0.36 |  |
|  |  |  |  |  |  | PKBS-internalizing behaviors | parent | 0.09 |  |
| Greenberg et al. (1991) | QED(S) | 308 students (136E, 172C) | NA; mean age 8.1 years; 30% special education; 45% ethnic minorities; USA | 1 year; 1,2 | high; 3 days per week; 35 lessons | KAI-appropriateness of examples of emotions: basic affects | task | 0.33 |  |
|  |  |  |  |  |  | KAI-appropriateness of examples of emotions: advanced affects | task | 0.00 |  |
|  |  |  |  |  |  | KAI-hiding feelings1 | task | 0.49 |  |
|  |  |  |  |  |  | KAI-hiding feelings2 | task | 0.38 |  |
| Greenberg et al. (1995) | QED(S) | 286 students (130E, 156C)  Special education:  94 students. (47E, 47C) | mid; USA | 1 year; 1,2 | high; 60 lessons; 3 times per week | feelings vocabulary | task | 0.61 | -0.09 |
|  |  |  |  |  |  | general feelings | task | 0.20 | 0.27 |
|  |  |  |  |  |  | emotion experience | task | 0.21 | 0.29 |
|  |  |  |  |  |  | recognize emotions | task | 0.16 | 0.46 |
|  |  |  |  |  |  | understanding feelings | task | 0.00 |  |
|  |  |  |  |  |  | emotion display rules | task | 0.18 |  |
|  |  |  |  |  |  | changing feelings | task | 0.03 |  |

| Goossens et al. (2012) | QED(L) | 18 schools (9E, 9C)  1294 students (674E, 620C) | NA; rural areas and provincial towns; Netherlands | 2 years;  K, 1,3,5 | Low; 40 lessons in total, but mean completeness was 0.50 and 0.49 | PBSI-ADH | teacher | 0.04 |  |
| --- | --- | --- | --- | --- | --- | --- | --- | --- | --- |
|  |  |  |  |  |  | PBSI-anxiety | teacher | -0.03 |  |
|  |  |  |  |  |  | PBSI-depression | teacher | -0.01 |  |
|  |  |  |  |  |  | SEQ-prosocial behavior | teacher | 0.06 |  |
|  |  |  |  |  |  | DDPC-total depression | student | -0.01 |  |
|  |  |  |  |  |  | PEER-social preference | peer | 0.02 |  |
|  |  |  |  |  |  | PKBS-social skills | teacher | 0.04 |  |
|  |  |  |  |  |  | HSCS-social emotional competence | teacher | 0.02 |  |
|  |  |  |  |  |  | LEAS-emotional awareness | task | 0.17 |  |
|  |  |  |  |  |  | DERS-emotion regulation | student | -0.03 |  |
|  |  |  |  |  |  | BEI-empathy | student | -0.10 |  |
| Hamre et al. (2012) | RCT(S) | 980 students (738E, 242C) | Low; at-risk children; class poor proportion 69%; USA | 8 months; pre | Low; 36 lessons; once a week | TCRS-social competence | teacher | 0.24 |  |
|  |  |  |  |  |  | TCRS-social problem behaviors | teacher | 0.10 |  |
| Hennessey & Humphrey. (2020) | RCT(L) | 45 schools (23E, 22C) 3287 students (1678E, 1609C) | Mid; 29.5% FSM; 21% EAL; UK | 2 years; 5,6 | Low; less than one lesson a week; high quality | Year 5 students: |  |  |  |
|  |  |  |  |  |  | InCAS-read/write | test | -0.02 | -0.01 |
|  |  |  |  |  |  | InCAS-math | test | -0.01 | 0.02 |
|  |  |  |  |  |  | Year 6 students: |  |  |  |
|  |  |  |  |  |  | KS2-english | test | -0.03 | 0.02 |
|  |  |  |  |  |  | KS2-maths | test | -0.05 | -0.03 |

| Hsueh et al. (2014) | RCT(S) | 28 centers (14E, 14C) 77 classrooms (37E, 40C) 446 students (226E, 220C) | NA; mean age 3.47, 50.65% female; USA | 1 year; pre | NA; conducted as "base curriculum" | BPI-behavior problem | teacher | 0.07 |  |
| --- | --- | --- | --- | --- | --- | --- | --- | --- | --- |
|  |  |  |  |  |  | SSRS-social skills | teacher | 0.22 |  |
|  |  |  |  |  |  | CFBRS-interpersonal skills | teacher | 0.00 |  |
|  |  |  |  |  |  | CFBRS-work-related skills (learning behaviors) | teacher | 0.19 |  |
|  |  |  |  |  |  | STRS-closeness | teacher | 0.16 |  |
|  |  |  |  |  |  | STRS-conflict | teacher | 0.00 |  |
|  |  |  |  |  |  | ARS-general knowledge | teacher | 0.06 |  |
|  |  |  |  |  |  | ARS-language and literacy | teacher | 0.11 |  |
|  |  |  |  |  |  | ARS-mathematical thinking | teacher | 0.05 |  |
| Humphrey et al. (2016) & (2018) | RCT(L) | 45 schools (23E, 22C) 4516 students (2340E, 2176C) | Mid; 29.7% FSM; UK | 2 years; 3,4,5 | low; 2 years; only delivered 20 of 40 lessons per year | SDQ-emotional symptoms | teacher | -0.1 | 0.23 |
|  |  |  |  |  |  | SDQ-conduct problems | teacher | -0.03 | -0.26 |
|  |  |  |  |  |  | SDQ-hyperactivity | teacher | -0.04 | 0.04 |
|  |  |  |  |  |  | SDQ-peer problems | teacher | -0.07 | 0.08 |
|  |  |  |  |  |  | SDQ-prosocial behaviors | teacher | 0.04 | 0.16 |
|  |  |  |  |  |  | SSIS-social emotional competence | student | -0.03 | 0.07 |
|  |  |  |  |  |  | KS27-psychological well-being | student | 0.12 |  |
|  |  |  |  |  |  | KS27-social support and peers | student | 0.11 |  |
|  |  |  |  |  |  | KS27-school environment | student | 0.03 |  |
|  |  |  |  |  |  | NPD-exclusions | NPD | 0.04 |  |
|  |  |  |  |  |  | NPD-attendance | NPD | 0.02 |  |
|  |  |  |  |  |  | NPD-reading/writing | NPD | -0.02 |  |
|  |  |  |  |  |  | NPD-math | NPD | 0.04 |  |

| Johannes (2003) | QED(S) | 5 sites (74E,44C) | NA, USA | 6 months; age 9-11 | low; 20-30 minutes once a week | social behaviors | teacher | 0.12 |  |
| --- | --- | --- | --- | --- | --- | --- | --- | --- | --- |
| Novak et al. (2017) | RCT(S) | 29 schools (14E, 15C) 568 students (280E, 288C) | NA; 47% girls; Croatia | 1.5 years; 1 | high; 63 lessons; two per week | SCS-prosocial behavior | teacher | 0.16 |  |
|  |  |  |  |  |  | SCS-emotional regulation | teacher | 0.18 |  |
|  |  |  |  |  |  | SRQ-learning behavior | teacher | 0.06 |  |
|  |  |  |  |  |  | ADHD-RS-inattention | teacher | 0.07 |  |
|  |  |  |  |  |  | ADHD-RS-hyperactivity | teacher | 0.12 |  |
|  |  |  |  |  |  | TOCA-oppositional behavior | teacher | 0.12 |  |
|  |  |  |  |  |  | TOCA-physical aggression | teacher | 0.11 |  |
|  |  |  |  |  |  | SDQ-peer problems | teacher | 0.00 |  |
|  |  |  |  |  |  | Head Start REDI-withdrawn behavior | teacher | 0.09 |  |
| Riggs et al. (2006) | QED(S) | 4 schools (2E, 2C) 318 students (153E, 165C) | NA; 4 schools in Seattle;55% C, 33% AA, 22% Asian A, Native A and other; USA | 9 months; 2,3 | high; taught 53 of 68 sessions; about 3 times per week | CWT-inhibitory control | task | 0.31 |  |
| Seyhan et al. (2019) | QED(S) | 42 classrooms (21E, 20C) 565 students (285E, 280C) | Mid; mainly served university personnel; Turkey | 3 months; pre | high; 33 lessons in 9 weeks | HSCS-social emotional competence | teacher | 0.42 |  |
|  |  |  |  |  |  | STRS-teacher student relationships | student | 0.29 |  |

| Sheard et al. (2012) & (2013) | QED(L) | 12 schools (6E, 6C)  aged 4–6: 37 classes, 673 students (283E, 390C)  aged 8–10:  37 classes, 791 students (359E,432C) | Mid; mainly served working class; 29%FSM; Northern Ireland | 2 years; 1,2,5,6 | NA | For 5-7 years students: |  |  |  |
| --- | --- | --- | --- | --- | --- | --- | --- | --- | --- |
|  |  |  |  |  |  | empathy, coping and co-operation | teacher | 0.37 |  |
|  |  |  |  |  |  | actively helps others | teacher | 0.51 |  |
|  |  |  |  |  |  | (lack of) negative affect | teacher | 0.27 |  |
|  |  |  |  |  |  | fighting and aggression | teacher | -0.01 |  |
|  |  |  |  |  |  | socially withdrawn | teacher | 0.00 |  |
|  |  |  |  |  |  | ACES-emotion recognition | student | 0.13 |  |
|  |  |  |  |  |  | FTI-managing emotions | student | 0.15 |  |
|  |  |  |  |  |  | For 9-11 years students: |  |  |  |
|  |  |  |  |  |  | empathy and co-operation | teacher | 0.22 |  |
|  |  |  |  |  |  | reflectivity and perseverance | teacher | 0.17 |  |
|  |  |  |  |  |  | fighting and aggression | teacher | 0.17 |  |
|  |  |  |  |  |  | negative affect | teacher | 0.07 |  |
|  |  |  |  |  |  | FTI-managing emotions | student | 0.12 |  |

| Social and Character Development Research Consortium. (2010) | RCT(S) | 10 schools (5E, 5C) 421 students (211E, 210C) | Low;47.7% FRL; 56% W, 22% B, 12% H, 10% other; USA | 3 years; 3-5 | low; 20-30 minutes per day, 3-5 days per week; half high fidelity | normative beliefs about aggression | student | 0.01 |  |
| --- | --- | --- | --- | --- | --- | --- | --- | --- | --- |
|  |  |  |  |  |  | empathy | student | -0.03 |  |
|  |  |  |  |  |  | altruistic behavior | student | 0.00 |  |
|  |  |  |  |  |  | altruistic behavior | parent | -0.06 |  |
|  |  |  |  |  |  | altruistic behavior | teacher | -0.31 |  |
|  |  |  |  |  |  | positive social behavior | parent | -0.08 |  |
|  |  |  |  |  |  | positive social behavior | teacher | 0.06 |  |
|  |  |  |  |  |  | problem behavior | student | -0.12 |  |
|  |  |  |  |  |  | problem behavior | parent | 0.04 |  |
|  |  |  |  |  |  | problem behavior | teacher | 0.14 |  |
|  |  |  |  |  |  | ADHD related behavior | teacher | 0.22 |  |
|  |  |  |  |  |  | engagement with learning | student | -0.11 |  |
|  |  |  |  |  |  | academic competence and motivation | teacher | 0.08 |  |
|  |  |  |  |  |  | positive school orientation | student | 0.00 |  |
|  |  |  |  |  |  | negative school orientation | student | 0.19 |  |
|  |  |  |  |  |  | student afraid at school | student | 0.22 |  |
|  |  |  |  |  |  | victimization at school | student | -0.20 |  |

| **Follow –up Study** | **Design/**  **Sample size** | **N** | **SES or Sample Characteristics** | **Duration** | **Dosage/ Implementation of PATHS** | **Outcomes** | **Evaluator** | **Study ES** | **Target**  **ed ES** |
| --- | --- | --- | --- | --- | --- | --- | --- | --- | --- |
| Averdijk et al. (2016) & Malti et al. (2011) | RCT(S) | 28 schools (14E, 14C) 716 students (360E, 356C) | Mid; mean ISEI 45; 55% Swiss nationality; public schools in Zurich, Switzerland | 5 years; 1-5 | high; high quality; 2.4 sessions per week | 3-year FU: |  |  |  |
|  |  |  |  |  |  | SBQ- aggressive behavior | teacher | 0.16 |  |
|  |  |  |  |  |  | SBQ-externalizing behavior-NACD | teacher | 0.04 |  |
|  |  |  |  |  |  | SBQ-externalizing behavior-ADHD | teacher | 0.15 |  |
|  |  |  |  |  |  | SBQ -aggressive behavior | parent | 0.12 |  |
|  |  |  |  |  |  | SBQ-externalizing behavior-ADHD | parent | 0.00 |  |
|  |  |  |  |  |  | SBQ- aggressive behavior | student | 0.08 |  |
|  |  |  |  |  |  | SBQ-externalizing behavior-ADHD | student | 0.08 |  |
|  |  |  |  |  |  | SBQ-prosocial behavior | teacher | -0.08 |  |
|  |  |  |  |  |  | SBQ-prosocial behavior | parent | -0.03 |  |
|  |  |  |  |  |  | SBQ-prosocial behavior | student | -0.08 |  |
|  |  |  |  |  |  | Problem solving-aggressive | task | 0.08 |  |
|  |  |  |  |  |  | Problem solving-socially competent | task | -0.04 |  |
|  |  |  |  | 7 and 9 years; 1-9 |  | 5-year FU: |  |  |  |
|  |  |  |  |  |  | Delinquency | student | 0.02 |  |
|  |  |  |  |  |  | police contact | student | 0.23 |  |
|  |  |  |  |  |  | substance use | student | 0.01 |  |
|  |  |  |  |  |  | peer aggression | student | -0.04 |  |
|  |  |  |  |  |  | SBQ-aggressive behavior | student | -0.10 |  |
|  |  |  |  |  |  | SBQ-prosocial behavior | student | -0.06 |  |
|  |  |  |  |  |  | aggressive conflict resolution | student | -0.04 |  |
|  |  |  |  |  |  | competent conflict resolution | student | -0.12 |  |
|  |  |  |  |  |  | substance use | teacher | 0.06 |  |
|  |  |  |  |  |  | deviance | teacher | 0.02 |  |
|  |  |  |  |  |  | SBQ-aggressive behavior | teacher | -0.05 |  |
|  |  |  |  |  |  | SBQ-prosocial behavior | teacher | -0.02 |  |
|  |  |  |  |  |  | SBQ-nonaggressive conduct disorder | teacher | -0.06 |  |
|  |  |  |  |  |  | 7-year FU: |  |  |  |
|  |  |  |  |  |  | Delinquency | student | 0.06 |  |
|  |  |  |  |  |  | police contact | student | 0.03 |  |
|  |  |  |  |  |  | substance use | student | -0.02 |  |
|  |  |  |  |  |  | peer aggression | student | -0.02 |  |
|  |  |  |  |  |  | SBQ-aggressive behavior | student | -0.07 |  |
|  |  |  |  |  |  | SBQ-prosocial behavior | student | 0.01 |  |
|  |  |  |  |  |  | aggressive conflict resolution | student | 0.02 |  |
|  |  |  |  |  |  | competent conflict resolution | student | 0.07 |  |
|  |  |  |  |  |  | substance use | teacher | 0.10 |  |
|  |  |  |  |  |  | deviance | teacher | 0.10 |  |
|  |  |  |  |  |  | SBQ-aggressive behavior | teacher | 0.10 |  |
|  |  |  |  |  |  | SBQ-prosocial behavior | teacher | -0.02 |  |
|  |  |  |  |  |  | SBQ-nonaggressive conduct disorder | teacher | 0.10 |  |

| Bierman et al. (1999) a & (2002) & (2004) & (2007) | RCT(S) | 54 schools 401 classrooms (191E, 210C) 891 students (445E, 446C) | mid; high-risk (top 10% on behavior problems); 35% lowest SES; 51% AA, 47% EA, 2% others; USA | 9 years;  1-9 | high; 2-3 sessions per week | 4-year FU: |  |  |  |
| --- | --- | --- | --- | --- | --- | --- | --- | --- | --- |
|  |  |  |  |  |  | SRD-antisocial behavior | student |  | 0.17 |
|  |  |  |  |  |  | proportion of receiving any externalizing psychiatric diagnosis(CD/ODD/ADHD) | interviewer |  | 0.12 |
| Humphrey et al. (2016) & (2018) | RCT(L) | 45 schools (23E, 22C) 4516 students (2340E, 2176C) | low; 29.4% FSM; UK | 2 years; 3,4,5 | low; 40 lessons per year; twice per week | 12-month FU: |  |  |  |
|  |  |  |  |  |  | KS27-psychological well-being | student |  | 0.05 |
|  |  |  |  |  |  | KS27-social support and peers | student |  | 0.02 |
|  |  |  |  |  |  | KS27-school environment | student |  | 0.04 |
|  |  |  |  |  |  | 24-month FU: |  |  |  |
|  |  |  |  |  |  | SSIS-social skills | student |  | -0.01 |
|  |  |  |  |  |  | SDQ-internalizing symptoms | teacher |  | 0.05 |
|  |  |  |  |  |  | SDQ-externalizing symptoms | teacher |  | 0.04 |
|  |  |  |  |  |  | SDQ-prosocial behavior | teacher |  | 0.15 |
|  |  |  |  |  |  | KS27-psychological well-being | student |  | -0.09 |
|  |  |  |  |  |  | KS27-social support and peers | student |  | -0.09 |
|  |  |  |  |  |  | KS27-school environment | student |  | -0.12 |
| Kam et al. (2004) | RCT(S) | 133 students (with special needs) | NA; students with special needs; 88W, 27AA, 18 other; USA | 3 years; 1,2 | high; 60 lessons; 3 times per week | CBCL-externalizing behaviors | teacher |  | 0.18 |
|  |  |  |  |  |  | CBCL-internalizing behaviors | teacher |  | 0.22 |
|  |  |  |  |  |  | TCRS-social competence | teacher |  | 0.00 |
|  |  |  |  |  |  | CDI-depression | student |  | 0.49 |
|  |  |  |  |  |  | KAI-feeling vocabulary | task |  | 0.41 |
|  |  |  |  |  |  | SPSI-social problem-solving skills | task |  | 0.00 |
| Riggs et al. (2006) | QED(L) | 4 schools (2E, 2C) 318 students (153E, 165C) | NA; 4 schools in Seattle;55% C, 33% AA, 22% Asian A, Native A and other; USA | 9 months; 2,3 | high; 53 lessons and 68 sessions; about 3 times per week | 1-year FU: |  |  |  |
|  |  |  |  |  |  | CBCL-externalizing behavior | teacher |  | 0.37 |
|  |  |  |  |  |  | CBCL-internalizing behavior | teacher |  | 0.25 |
